# Development and Dissemination of the Japanese Translation of the Mammalian Phenotype Ontology as an Open Resource

**DOI:** 10.1002/cga.70070

**Published:** 2026-07-03

**Authors:** Terue Takatsuki, Susan M. Bello, Tatsuya Kushida, Eisuke Dohi, Atsushi Yoshiki, Toyofumi Fujiwara, Susumu Goto, Hiroshi Masuya

**Affiliations:** ^1^ Database Division for Life Science, BioData Science Initiative National Institute of Genetics, Research Organization of Information and Systems Kashiwa Chiba Japan; ^2^ The Jackson Laboratory Bar Harbor Maine USA; ^3^ RIKEN BioResource Research Center Tsukuba Ibaraki Japan; ^4^ National Institute of Neuroscience NCNP Kodaira Tokyo Japan

**Keywords:** biological ontologies, human‐in‐the loop translation, large language model, natural language processing, phenotype

## Abstract

The Mammalian Phenotype Ontology is a comprehensive controlled vocabulary encompassing phenotypic terms related to congenital anomalies, developmental abnormalities, and other mammalian phenotypes, developed by Mouse Genome Informatics. Although this ontology was originally created in English, multilingualization is essential for broadening its impact by improving accessibility for researchers worldwide. To address this need, we developed a Japanese translation of the Mammalian Phenotype Ontology to support non‐English‐speaking research communities in Japan. The Japanese translation of this ontology was produced using a systematic human‐in‐the‐loop workflow consisting of machine translation, systematic manual review, and expert curation. Manual curation is a critical component of this process, as automated translation alone is insufficient for accurately rendering domain‐specific phenotype terminology. The Japanese‐translated Mammalian Phenotype Ontology has been integrated into multiple data resources and search platforms, including experimental animal resource databases and disease‐oriented data integration services. This integration enables users to search for and interpret annotated mouse phenotypes using Japanese terminology and has significantly improved the accessibility and usability of Mammalian Phenotype Ontology‐based services for non‐English‐speaking Japanese researchers, students, and laboratory animal caretakers. In addition, the Japanese translation of the Mammalian Phenotype Ontology is publicly released and formally incorporated into the official Mammalian Phenotype Ontology distribution through collaboration with Mouse Genome Informatics, allowing regular updates synchronized with ongoing development. This study highlights the importance of localizing ontology vocabularies to advance global accessibility and presents a practical framework for systematic ontology translation.

## 1 Introduction

The Mammalian Phenotype Ontology (MP) [1] is a comprehensive ontology describing phenotypes observed in mammals and provides standardized terminology for phenotypic abnormalities relevant to congenital anomalies and developmental disorders. Developed at The Jackson Laboratory in the United States, MP was constructed by systematically extracting phenotypic information from mouse‐based research and assigning unique identifiers to each term. As of January 2026, MP contains 14 451 terms, with approximately 200 new terms added annually. MP is widely used for ontology‐based annotation of mouse and rat phenotypes by major biological databases, including Mouse Genome Informatics (MGI) [2], the Rat Genome Database (RGD) [3], and the International Mouse Phenotyping Consortium (IMPC) [4].

Although many biomedical ontologies are developed and maintained primarily in English, language barriers can limit their effective adoption in research, education, and clinical practice. To address this challenge, internationalization efforts for the Human Phenotype Ontology (HPO) [5] were initiated at an early stage, with the goal of facilitating global collaboration and improving accessibility for non‐English‐speaking communities. The Japanese translation of HPO [6] has since been widely adopted in a range of applications, including web services that support rare and genetic disease diagnosis based on clinical case reports [7] and data‐exchange platforms [8] for genetic variants associated with undiagnosed diseases. These efforts demonstrated that localized phenotype ontologies can substantially enhance accessibility, usability, and community participation in phenotype‐driven research.

Building on the success of HPO internationalization, our collaborative research group in Japan recognized the need to localize MP to support phenotype‐based research and data utilization by non‐English‐speaking communities. Approximately a decade ago, we initiated the Japanese translation of MP, and through sustained and systematic curation efforts, completed all translations, totaling more than 14 000 MP terms by 2023. Japanese was the first language incorporated into the MP internationalization framework. This effort was conducted in close collaboration with MGI, resulting in the public release of the Japanese language profile as part of the MP International Edition [9].

While recent advances in machine translation have enabled rapid generation of multilingual content, such approaches remain insufficient for translating domain‐specific biomedical ontologies. Phenotype ontologies contain specialized terminology, compound expressions, and context‐dependent descriptors that often cannot be accurately rendered through automated translation alone. In particular, subtle distinctions in developmental and congenital anomaly‐related phenotypes require expert interpretation to ensure semantic accuracy and practical usability. Therefore, manual inspection and expert curation play a critical role in producing reliable localized ontology resources.

In this paper, we present a systematic framework for ontology localization, exemplified by the Japanese translation of MP, and highlight the importance of expert curation for ensuring semantic accuracy and global accessibility of phenotype‐based resources.

## 2 Materials and Methods

To localize MP while preserving semantic accuracy and practical usability, we established a systematic human‐in‐the‐loop translation framework. This framework combines automated machine translation for scalability with systematic manual review and expert curation to address the limitations of fully automated approaches, particularly for domain‐specific phenotype terminology related to developmental abnormalities and congenital anomalies.

### 2.1 Overview of the Human‐In‐The‐Loop Translation Framework

The Japanese translation of MP was developed using a systematic human‐in‐the‐loop translation framework designed to ensure semantic accuracy and domain appropriateness of phenotype terminology. In this framework, machine translation is used to generate preliminary translation candidates, which are then systematically reviewed, revised, and validated through manual curation and expert evaluation as core components of the translation process.

The initial full Japanese translation of MP, completed by March 2023, was produced by applying this framework to all available MP terms obtained from the MGI website [10]. Human oversight played a critical role in correcting domain‐inappropriate expressions produced by automated or general‐purpose translation. For example, human‐centric translations such as “male” rendered as “男性” (man) and “female” as “女性” (woman) were manually revised to animal‐appropriate terms, “雄” (male) and “雌” (female), respectively. These corrections illustrate the necessity of human judgment in translating phenotype ontology terms for experimental animal research.

Since March 2023, the same human‐in‐the‐loop framework applied monthly to maintain synchronization with ongoing MP development. Importantly, this framework is applied not only during the initial full translation of the ontology but also during routine monthly updates, allowing continuous integration of newly added or modified MP terms.

**FIGURE 1:**
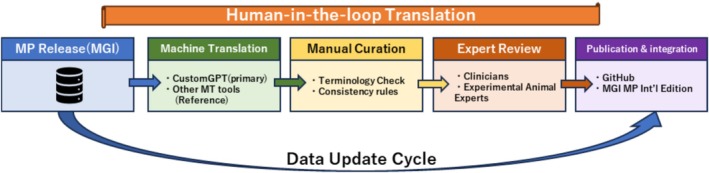
The systematic human‐in‐the‐loop translation workflow. The workflow consists of five standardized steps integrated into a monthly update cycle. (1) Updated MP terms are retrieved from MGI. (2) Initial translation candidates are generated using CustomGPT, guided by a domain‐specific glossary. (3) Curators perform manual checks for terminological consistency. (4) Clinical and biological experts review the terms for accuracy. (5) The validated translations are published and synchronized with the official MP international edition.

This operational workflow consists of five standardized steps: (1) download and verification of updated MP files, (2) machine translation of newly added or modified terms, (3) systematic manual curation, (4) expert review, and (5) publication of the translated data (see Figure 1). The details of each step are described in the sections below.

### 2.2 Source Data and Scope of Translation

The source data for the Japanese translation were obtained from official MP releases [10] provided by MGI. All non‐obsolete MP terms were translated, with monthly updates applied to newly added or modified terms to remain synchronized with ongoing MP development. While those that became obsolete after translation were removed from the distributed Japanese dataset, they were retained separately as obsolete terms.

### 2.3 Initial Machine Translation

In the initial stage of the translation workflow, machine translation was used solely to generate candidate translations and to capture the general meaning of newly introduced terms. At the early phase of this work, general‐purpose machine translation services, including Google Translate [11] and DeepL [12], were used to obtain an overview of candidate translations.

With the subsequent emergence of large language models, we examined their applicability to MP translation tasks. However, the quality of translations generated by general‐purpose large language models was largely comparable to that of existing machine translation services and remained insufficient for reliably translating domain‐specific phenotype terminology.

To address this limitation, we employed a customized version of a large language model (CustomGPT) specifically configured for MP translation [13]. The customization consisted of a prompt‐based translation workflow in which a domain‐specific glossary curated by the Terminology Committee of the Japanese Teratology Society [14] was supplied as a structured reference at inference time, without retraining the underlying model. This glossary provides standardized Japanese terminology for congenital anomalies and developmental abnormalities, enabling more appropriate initial translations of phenotype terms relevant to experimental animal and developmental research.

This reference‐based approach enabled the model to generate terminology‐guided translation candidates by constraining candidate generation to established domain terminology, while preserving flexibility for subsequent human curation.

At present, CustomGPT serves as the primary tool for generating translation candidates, while other machine translation services are consulted selectively when additional reference or comparison is needed. Nevertheless, all machine‐generated translations were treated strictly as preliminary candidates and were subjected to comprehensive manual curation and expert review to ensure semantic accuracy and practical usability. This approach ensured that improvements in automated translation enhanced efficiency while preserving the central role of human expertise in determining the final translations.

### 2.4 Manual Curation of Translated Terms

Following the initial machine translation, systematic manual curation was performed as a central step of the human‐in‐the‐loop framework to ensure semantic accuracy and consistency of the translated phenotype terms. Each candidate translation was evaluated to determine whether it was appropriate as life science terminology, with reference to specialized dictionaries, relevant online resources, and literature searches. In addition, curators examined the correspondence between the original MP term definitions and their Japanese translations to ensure conceptual alignment.

When machine translation outputs were consistent across tools, this consistency was taken as an indication of an established Japanese term. For example, in the case of MP:0030614 methylmalonic aciduria, all machine translation results were identical, and the consistent translation was, therefore, adopted (Table 1).

**TABLE 1:** Comparison of machine translation outputs for “MP:0030614 methylmalonic aciduria”.

Translation method	English label	Japanese translation	Notes
Google	Methylmalonic aciduria (MP:0030614)	メチルマロン酸尿症	Methylmalonic aciduria (メチルマロン酸尿症) is classified as one of the designated intractable diseases in Japan.
DeepL		メチルマロン酸尿症	
ChatGPT		メチルマロン酸尿症	

*Note:* Japanese translations generated by three machine translation systems.

In contrast, when discrepancies were observed among machine translation outputs, curatorial judgment was required to select an appropriate translation. For example, for “MP:0031543 abnormal heart ventricle size,” the translation “心室サイズの異常” was chosen (Table 2). During the curation process, recurring linguistic patterns were identified and standardized. Specifically, when adjectives such as “abnormal” appeared at the beginning of multiple English terms with similar semantic structure, a consistent translation strategy was applied by placing the corresponding Japanese expression at the end of the phrase. In this case, translating “abnormal” as “異常” at the end of the Japanese term helped maintain consistency across related phenotype expressions.

**TABLE 2:** Comparison of Japanese translations for “MP:0031543 Abnormal Heart Ventricle Size” generated by different translation methods.

Translation method	English label	Japanese translation	Notes
Manual (expert)	Abnormal heart ventricle size (MP:0031543)	心室サイズの異常	Natural and standard expression; semantically accurate and suitable as a preferred label.
Google		異常な心室サイズ	Clear in meaning, but not suitable as a controlled term.
DeepL		心室の大きさの異常	Semantically correct, but descriptive rather than terminological.
ChatGPT		心室サイズの異常な大きさ	Semantically correct, but descriptive rather than terminological.
CustomGPT		心室サイズ異常	Highly searchable, but semantically ambiguous.

### 2.5 Expert Review by Domain Specialists

Following manual curation, the translated terms were reviewed by domain specialists, including one clinical physician with expertise in laboratory animal science, three laboratory animal professionals, and one genetics specialist. This qualitative expert review, conducted by specialists with representative expertise in medicine, genetics, congenital anomalies, and laboratory animal research, ensured terminological accuracy and interpretability in both clinical and experimental contexts. Expert feedback was particularly important for distinguishing clinically or biologically meaningful differences that could not be reliably resolved through automated translation alone. For example, the terms “MP:0013752 stridor” and “MP:0031086 wheezing” were initially assigned the same machine‐translated Japanese term (“喘鳴”). Even after manual curation, this single translation remained insufficient to distinguish these clinically distinct respiratory sounds. At the expert review stage, the translations were, therefore, differentiated by appending the original English terms in parentheses to preserve clarity and accuracy. Accordingly, MP:0013752 was translated as “喘鳴 (stridor)” and MP:0031086 as “喘鳴 (wheezing)”.

Similarly, the terms “MP:0000756 *forelimb paralysis*” and “MP:0031203 *forelimb paresis*” were both initially translated as “前肢麻痺” by machine translation. Based on expert evaluation of the difference in severity between paralysis and paresis, the final translations were revised to “前肢麻痺” for *forelimb paralysis* and “前肢筋力低下” for *forelimb paresis*.

These examples demonstrate that expert review is essential for preserving clinically and biologically meaningful distinctions in phenotype terminology and for ensuring the practical usability of translated ontology resources.

### 2.6 Quality Control and Consistency Assurance

To ensure the reliability and long‐term usability of the Japanese translation of MP, quality control was implemented as an integral component of the human‐in‐the‐loop translation framework rather than as a single post hoc step. Multiple layers of review were applied throughout the workflow to maintain semantic accuracy, terminological consistency, and practical usability.

Consistency of translated terminology was systematically evaluated across related MP terms. Particular attention was paid to recurring linguistic patterns, such as the placement of modifiers, expressions of abnormality, and severity‐related distinctions, to avoid inconsistencies that could impair interpretation or searchability. When discrepancies were identified, translations were revised to conform to shared conventions established during the curation process.

Feedback from domain experts was incorporated iteratively, and discrepancies between curatorial and expert interpretations were resolved through discussion and consensus building. This iterative review process contributed to coherence across the translated vocabulary and ensured that the final translations were suitable for both computational use and human interpretation.

### 2.7 Publication and Maintenance Strategy

The finalized Japanese translation of MP is publicly released to ensure broad accessibility and reuse. The translated data is made available through a version‐controlled repository [15], enabling integration with external systems and downstream applications. To support long‐term maintenance, the translation workflow is aligned with the official MP release cycle. Newly added or modified terms are reviewed and incorporated monthly, ensuring that the Japanese translation remains synchronized with ongoing MP development. This maintenance strategy supports consistent availability of up‐to‐date translated phenotype terminology for research and data annotation purposes.

## 3 Results

### 3.1 Overview of the Japanese MP Translation Resource

The Japanese translation of MP was established through multiple trial phases before the current translation workflow was finalized. In March 2023, a complete Japanese translation of the MP vocabulary, consisting of approximately 14 000 terms, was publicly released. As of January 2026, the translated vocabulary has expanded to 14 451 terms through continuous monthly updates synchronized with official MP releases. To maintain translation quality during these ongoing updates, routine manual curation has been performed following automated translation. Through routine curation, a substantial proportion of newly added or modified MP terms were found to require manual revision after machine translation. In recent updates, approximately 80% of newly introduced or modified terms required some level of human correction to ensure semantic accuracy and domain appropriateness (Table 3). On average, each update involved 50–100 new or modified terms. The translation and curation process is primarily conducted by one curator and one medical doctor with expertise in laboratory animal science. When necessary, additional domain experts are consulted to resolve complex or ambiguous cases. This workflow enables efficient handling of approximately 200 newly added terms per year while maintaining high semantic accuracy.

**TABLE 3:** Breakdown of translation outcomes for newly added MP terms (*n* = 138) between May 7, 2025 and January 9, 2026.

Finalization stage outcome	Number of terms	Percentage of newly added terms (%)
Machine translation accepted	23	17% (23/138)
Manual curated accepted	43	31% (43/138)
Expert curated—medical terminology	15	10% (15/138)
Expert curated—Japanese particles position change	57	41% (57/138)

*Note:* The values represent the number and percentage of newly added terms (*n* = 138) whose translations were finalized at each stage.

### 3.2 International Integration Through Collaboration With MGI

The Japanese translation of MP was developed in close collaboration with MGI and has been formally incorporated into the MP International Edition. As a result, the Japanese language profile is distributed alongside the primary MP release through the MGI website [16], ensuring consistent availability and regular updates synchronized with official MP development.

**FIGURE 2:**
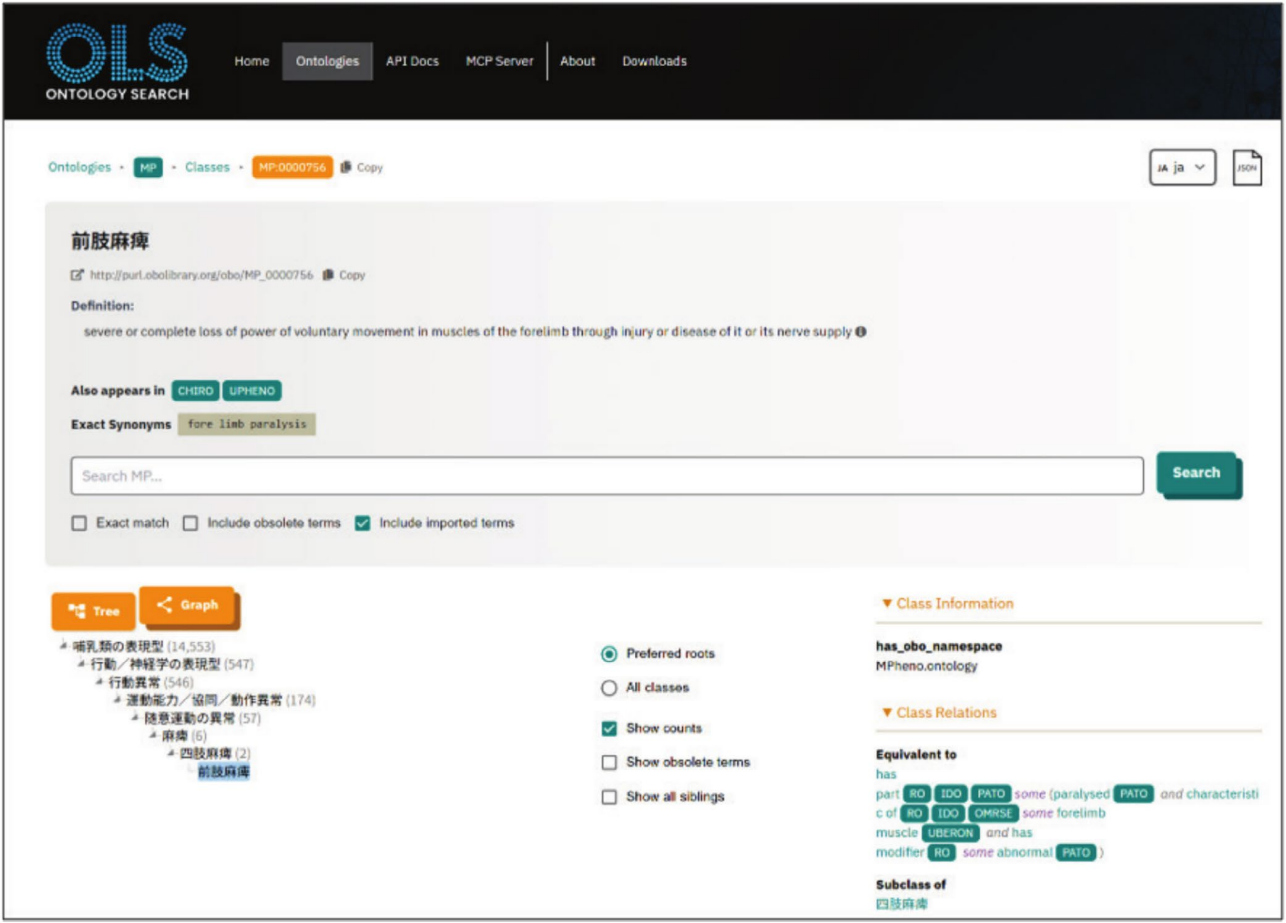
Display of the Japanese translation in the Ontology Lookup Service (OLS). The screenshot shows the detailed view of an MP term (e.g., MP:0000756 forelimb paralysis). The Japanese translation is displayed alongside the English label, definition, and synonyms. Integration into OLS enables users to search for and browse Mammalian Phenotype Ontology terms using Japanese queries. *Source:* Image captured from the OLS website [17].

This integration demonstrates that the Japanese translation is not an isolated local resource but a formally recognized component of the official MP distribution. The integrated MP files [9] serve as data sources for multiple international life science information systems, enabling access to MP terminology in Japanese. For example, users can browse and search MP terms in Japanese via the Ontology Lookup Service (OLS) [17] operated by the European Bioinformatics Institute (EMBL‐EBI) (Figure 2).

### 3.3 Application to Experimental Animal Resource Search (RIKEN BRC)

The availability of Japanese MP translations has significantly improved the usability of experimental animal resource databases in Japan. At the RIKEN BioResource Research Center (RIKEN BRC), MP terms in both English and Japanese are used to annotate phenotypic information for mouse strains, enabling accurate interpretation across a wide range of experimental and research contexts.

**FIGURE 3:**
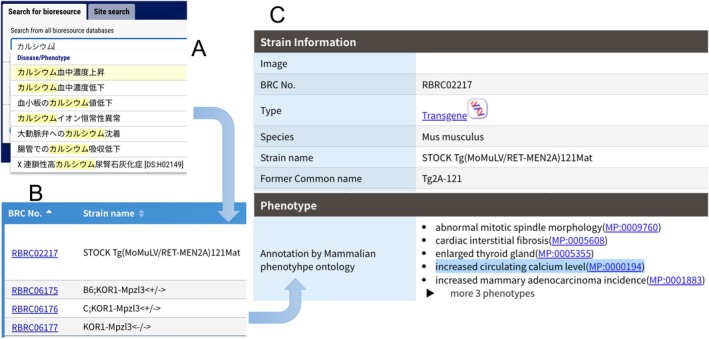
Search workflow of the bioresource search system using Japanese phenotype terms. (A) When the user inputs a keyword such as “カルシウム” (calcium), the system suggests phenotype terms that partially match the input (e.g., “カルシウム血中濃度上昇”, increased circulating calcium level). (B) Upon selecting the term, the system lists all mouse strains (BRC No.) annotated with the selected phenotype. (C) By selecting a specific BRC No. (e.g., RBRC02217), the user can view detailed information about the corresponding mouse strain. *Source:* Reproduced from the RIKEN BRC website [18].

The Japanese translations are integrated into RIKEN BRC's search systems [18], including “Search for Bioresources” and “Advanced Search” (Figure 3), allowing users to search for mouse strains using either English or Japanese MP terms. This bilingual search capability enables researchers to identify mouse strains that match their specific experimental objectives and phenotype requirements, particularly benefiting users who are less familiar with English phenotype terminology.

### 3.4 Application to Disease‐Oriented Data Integration (NanbyoData)

The Japanese translation of MP has also been applied to disease‐oriented data integration in NanbyoData [19], a platform that aggregates information on rare and intractable diseases in Japan. NanbyoData presents potential research resources, including experimental animal models, linked to diseases through gene associations.

Within NanbyoData, mouse strains relevant to specific diseases are displayed together with their annotated MP terms, which are shown in Japanese. This enables users to interpret phenotype information of animal models in their native language, facilitating understanding of how experimental mouse phenotypes relate to human disease characteristics.

**FIGURE 4:**
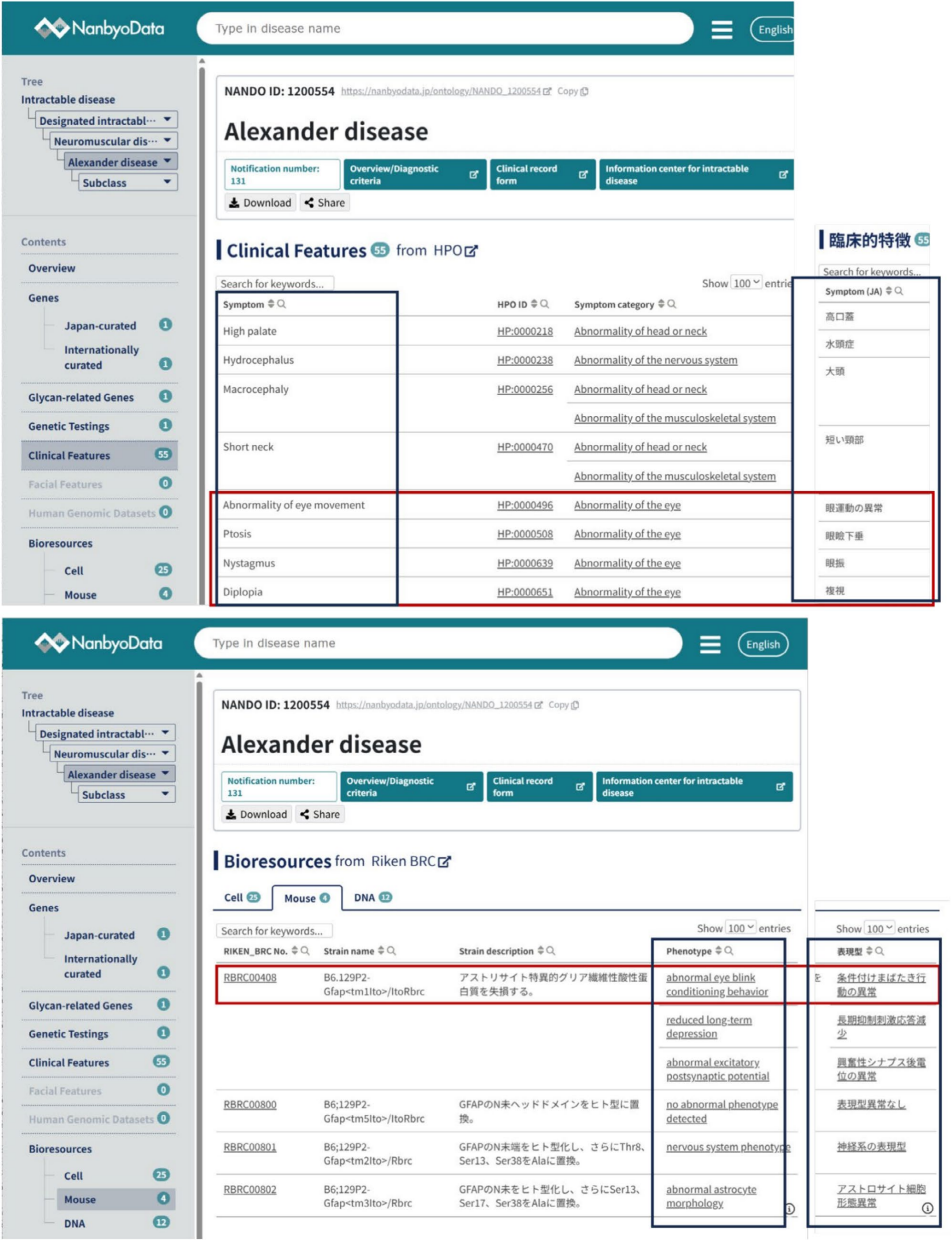
Integrated phenotype presentation in NanbyoData using HPO and MP. Clinical features of a disease are displayed using HPO terms with corresponding Japanese and English labels (blue boxes). Mouse strains associated with the disease are listed in the Bioresources section and annotated with MP terms, for which Japanese translations are also provided (blue boxes). Red boxes highlight phenotypes with semantic correspondence between HPO‐based clinical features and MP‐based mouse phenotypes. *Source:* Reproduced from the NanbyoData website [19].

In addition, NanbyoData provides Japanese translations of HPO, allowing users to compare human disease phenotypes and mouse model phenotypes side by side in Japanese. As shown in Figure 4, clinical features of diseases are presented using HPO terms, while phenotypes of mouse strains are displayed using MP annotations, with Japanese and English labels provided for both. This bilingual and cross‐species phenotype presentation supports more intuitive interpretation of phenotype similarities and differences, thereby enhancing the utility of phenotype‐based data exploration for disease research (Figure 4).

## 4 Discussion

While most biomedical ontologies are originally developed in English, localization into additional languages is essential for improving accessibility and practical usability across diverse research communities. Several localization efforts have demonstrated the benefits of providing ontology resources in Japanese, such as the Japanese translation of the Mondo disease ontology [20], which has enhanced medical workflows by enabling disease term searches in Japanese.

However, the demonstrated value of localization raises an important methodological question: how can such ontology resources be translated accurately and sustainably into other languages? Our study highlights the limitations of relying solely on automated translation when localizing biomedical ontology vocabularies, particularly in specialized domains where precise interpretation and contextual understanding are required. In such cases, automated translation alone is often insufficient to capture domain‐specific nuances and conventions reliably.

A central challenge encountered during localization was that semantically similar MP terms were often translated into identical Japanese expressions by automated translation, obscuring important conceptual distinctions. To resolve this ambiguity, definitions were carefully examined and relevant scientific literature was consulted to assign distinct and accurate translations. A second challenge arose from discrepancies between human clinical terminology and experimental animal terminology. While clinical expressions are commonly used to describe human disease phenotypes, direct application of such terms to animal‐specific findings can be misleading. Systematic expert curation was, therefore, required to ensure that translations appropriately reflected the biological and experimental context of animal phenotypes. A third challenge involved outdated expressions and inconsistencies in Japanese kanji usage, particularly following the 2010 revision of commonly used characters in Japanese. In these cases, archaic terms were updated to modern equivalents to improve readability and usability, while legacy notations were retained where necessary to maintain compatibility with older literature. A fourth challenge concerned the placement of Japanese particles, where different choices of particle position can shift the semantic focus of a translated term. Because particle usage affects which element of a phrase is emphasized, no single solution can be considered universally correct. This issue remains unresolved and represents an ongoing topic for further examination in ontology localization. Together, these challenges underscore that ontology localization is not merely a technical translation task but a process that requires systematic manual curation, domain‐specific knowledge, and linguistic expertise. Although these issues were identified in the context of English‐to‐Japanese translation, similar or related challenges are likely to arise when localizing biomedical ontologies into other languages.

One of the most significant challenges in the human‐in‐the‐loop translation workflow is the time required for manual curation. The process of reviewing AI‐generated translations and subsequently validating them through expert evaluation is inherently labor‐intensive, as each term must be carefully examined to ensure semantic accuracy and domain appropriateness. Furthermore, because expert review cannot be fully automated, the workflow is often influenced by the availability and scheduling of domain specialists.

Although we have explored the potential of AI‐assisted evaluation of translation outputs, current approaches remain insufficient for reliably replacing expert judgment. Improving the efficiency of multilingual ontology translation will, therefore, require more advanced AI systems capable of effectively incorporating domain‐specific knowledge.

In the future, it may be possible to develop language‐agnostic AI agents that can extract relevant domain knowledge, generate multiple candidate labels from different perspectives, and provide objective justifications for label selection based on factors such as domain‐specific usage frequency. Such systems could substantially reduce the burden of manual curation while maintaining high semantic accuracy.

In addition to these challenges, sustainability is another critical aspect of ontology localization efforts. Biomedical ontologies such as MP are continuously evolving, with new terms added and existing definitions refined over time. Without mechanisms for regular updates, localized translations risk becoming outdated and losing practical value. In this context, international collaboration plays a critical role in ensuring long‐term maintenance and consistency of localized ontology resources. The formal incorporation of the Japanese MP translation into the official MP distribution through collaboration with MGI enables synchronization with ongoing ontology development and ensures that updates are propagated reliably across languages. This collaborative framework illustrates that sustainable ontology localization requires not only technical solutions but also coordinated governance and shared responsibility among international partners.

The integration of the Japanese MP translation into experimental animal resource databases and disease‐oriented data platforms demonstrated the practical impact of localization. At resources such as RIKEN BRC and NanbyoData, Japanese MP terms enabled users to search for and interpret phenotype annotations more intuitively, facilitating accurate selection of animal models aligned with specific research objectives. These outcomes illustrate that localization directly influences the efficiency and quality of phenotype‐based data utilization.

Beyond local usability, the Japanese translation of MP contributes to broader efforts in cross‐species phenotype interpretation and international collaboration. Localization not only improves human comprehension but also supports the computational handling of phenotypic data through stable identifiers, thereby enhancing research efficiency in experimental planning and strain selection [21]. In addition, the use of Japanese, including kanji, facilitates intuitive understanding of complex phenotype concepts and promotes broader engagement with ontology‐based resources.

The availability of Japanese translations for both MP and the HPO enables side‐by‐side comparison of human disease phenotypes and animal model phenotypes within a shared linguistic framework. Building on the MP localization effort, we also contributed to the Japanese translation of HPO and examined terminology consistency between the two ontologies [22]. This shared representation supports more intuitive interpretation of phenotypic similarities and differences across species and enhances the practical utility of phenotype‐based data integration. At the same time, inherent differences between human clinical descriptions and experimental animal phenotypes highlight the need for systematic mapping strategies and shared translation guidelines to ensure consistent cross‐species interpretation [23]. Addressing these issues will require coordinated curation strategies and continued refinement of cross‐ontology mappings.

Taken together, this work demonstrates that localization of biomedical ontologies yields benefits beyond language accessibility, including improved data interoperability, enhanced annotation sharing with international partners, and increased participation of non‐English‐speaking research communities. As a case study of ontology localization in Japan, it highlights key considerations for similar efforts in other languages and underscores the value of sustained investment in multilingual ontology development.

## 5 Conclusion

This study presents a systematic human‐in‐the‐loop framework for localizing MP into Japanese. By combining automated translation with expert curation, the use of a domain‐specific terminology resource curated by the Japanese Teratology Society, and international collaboration, we demonstrate a sustainable approach to ontology localization that preserves semantic accuracy. The Japanese translation of MP provides a practical model for multilingual ontology development and supports broader accessibility of phenotype‐based data resources.

## Funding

This work was supported in part by ROIS‐DS‐JOINT (034RP2023) awarded to T. Kushida. The authors also acknowledge support from the NBDC Program of the Japan Science and Technology Agency (JST).

## Ethics Statement

This study does not involve human participants, human data, or animals. Therefore, ethical approval and informed consent were not required.

## Consent

The authors have nothing to report.

## Conflicts of Interest

The authors declare no conflicts of interest.

## Data Availability

The Mammalian Phenotype Ontology (MP) is publicly available from the Mouse Genome Informatics (MGI) database. The Japanese translation developed in this study is openly available at https://github.com/dbcls/MP_Japanese, and will be continuously updated to reflect new ontology releases.
